# Subclinical mastitis: Prevalence and risk factors in dairy cows in East Java, Indonesia

**DOI:** 10.14202/vetworld.2021.2102-2108

**Published:** 2021-08-16

**Authors:** Himmatul Khasanah, Hidayat Bambang Setyawan, Roni Yulianto, Desy Cahya Widianingrum

**Affiliations:** Department of Animal Husbandry, Faculty of Agriculture, University of Jember, Jl. Kalimantan No 37, Jember 68121, Indonesia

**Keywords:** California mastitis test, lactating dairy cows, management practice, subclinical mastitis

## Abstract

**Background and Aim:**

Subclinical mastitis (SCM) is a disease that frequently attacks lactating dairy cows and possibly decreases production, causing economic losses to farmers. This study aimed to define the prevalence of SCM and risk factor-associated related management practice by dairy farmers in East Java, Indonesia.

**Materials and Methods:**

The milk sample per quarter of individual lactating Friesian–Holstein cows (592 samples) was collected from 148 cows from 62 selected farms in the region with high dairy cattle populations in Malang, Sidoarjo, Mojokerto, Probolinggo, Pasuruan, Lumajang, Jember, and Banyuwangi. SCM determination was performed using the California mastitis test. A survey including field observation and interviews with farmers was conducted to find out the management practices of the selected farms. The analysis of risk factors was conducted by multinomial regression using the IBM SPSS version 26.0 (IBM SPSS Statistics, Chicago, USA).

**Results:**

In addition, 68.18% and 66.72% at the cow and quarter levels, respectively, of the lactating dairy cows examined had SCM. In multinomial regression, four variables were significantly given impact in association with the SCM prevalence in identifying related lactation stage (odds ratio [OR]: 1-2 months=5.67, 2-6 months=9.435), teat wiping after milking (OR=42.197), house cleanliness (OR: dirty=0.120, moderate=0.527), and location (regencies) of raising the cows (OR: Sidoarjo=0.076, Mojokerto=0.165, Jember=1.210, Probolinggo=3.449, Lumajang=1.638, Malang=1.210, and Pasuruan=0.681).

**Conclusion:**

The SCM prevalence in East Java is relatively high and a threat to the dairy industry’s performance. This study found a significant association with SCM that needs to be considered in the practice of management to prevent and control SCM. However, the finding also suggested that hygienic management practices performed by farmers need to be improved to reduce SCM incidents.

## Introduction

One of the large milk-producing provinces in Indonesia is East Java. A production increment has been noted, but it has not been able to keep up with the increasing national demand for milk [1]. Maintaining the quantity and quality of milk produced by livestock is very important, where the selection of the superior breed can produce a large quantity and good milk quality [2]. The environmental factors in the form of feed and practical management in the sustaining system also contribute to dairy cows’ production [3]. Decreasing milk productivity in lactating dairy cows is influenced by several factors (e.g., breed, health, disease, feed, and management practice) [4]. Mastitis is one of the diseases that often attack dairy cows and reduce milk production [5,6]. Inflammation in the udder due to mastitis could be categorized into two: Clinical mastitis showing variation in milk (e.g., color change, clots, consistency, and lowered production) and inflammation symptoms in the udder. Even cattle show dehydration, hyperthermia, and lethargy [7]. Meanwhile, subclinical mastitis (SCM) is a type where no visible inflammation is noted and is asymptomatic but can reduce milk production [8].

SCM will affect the somatic cell count of ≥200,000 cells/mL, indicating chronic SCM [9]. Furthermore, aside from reducing milk production, mastitis also reduces the amount of casein, the percentage of lactose, and the total protein [8]. The decline in milk production could be approximately 2.6-43.1%, which is a problem for the milk industry in Indonesia and the global dairy industry. Economically, mastitis can cause losses with an average failure cost of $121-$131 per cow [10,11]. The economic losses can be due to reduced production (66%), discarding of milk (5.7%), replacement cost (22.6%), extra labor (0.1%), treatment (4.1%), and veterinary service (1.5%) [12]. Mastitis incidence caused by microorganisms from the bacteria (mostly *Staphylococcus aureus*, non*-S. aureus*, *Mycoplasma* spp., *Corynebacteriumbovis*, *Escherichia coli*, and *Klebsiella* spp.); fungi (*Penicillium* spp., *Aspergillus* spp., *Alternaria* spp., *Fusarium* spp., *Absidia* spp., and *Aureobasidium pollulans*), yeast (*Candida etchellsii*, *Candida albicans*, *Candida guilliermondii*, *Candida parapsilosis*, *Candida kefyr*, *Candida famata*, *Candida krusei*, *Chrysosporium* spp., *Geotrichum candidum*, *Trichosporon* spp., *Rhodotorula* spp., and *Saccharomyces cerevisiae*), and algae (mostly *Prototheca zopfii* and in small amounts of *Prototheca wickerhamii*) [13-16]. The incidence of SCM in Indonesia and several developing countries is relatively high, that is, Central Java (46.41%), Boyolali Indonesia (65%) [17,18], Iran (67.20%) [14], Rwanda and North-West Ethiopia (62%) [19], and Kenya (50.9%) [20]. The incidence of SCM in East Java was reported to be approximately 70% in 2018 and increased to 85% in 2019 [21-23]. The prevalence of SCM incidents in regions that produced milk has to be updated to detect mastitis diseases.

This study aimed to investigate the prevalence of SCM and identify the risk factors that caused SCM in dairy farms in East Java Province. This information can be used as a reference for designing prevention and management strategies and reducing the incidence of mastitis.

## Materials and Methods

### Ethical approval

Ethical approval was not necessary for this study as the milk samples collected from lactating dairy cows and the milking was carried out by each farmer according to their operational procedures.

### Study period and area

This study was conducted in September and October 2020. Milk samples were obtained from lactating dairy Friesian–Holstein (FH) cows, and each farmer carried out milking following their operational procedures. The SCM incidence survey was conducted through a purposive sampling method in several districts in East Java that are known to produce cow’s milk (i.e., Jember, Lumajang, Banyuwangi, Probolinggo, Pasuruan, Mojokerto, Sidoarjo, and Malang Regencies. Some farming systems were independent (individual farmers) or cooperative businesses (in groups).

Farms with the highest population in each area were selected. Among the districts observed, the sample from Malang Regency, the largest milk producer in East Java, was collected from the Pujon District. These farmers were members of the Tirtasari Gemilang Business Group and mainly carry out raising cattle as a side business to support their primary business as rice or vegetable farmer. Milk samples from Sidoarjo Regency were obtained from three locations (i.e., Karang Puri Village, Wonoayu District; Ploso Village, Wonoayu District; and Cemengkalang Village, and Sidoarjo District). Milk samples from the Pasuruan Regency were obtained from KUD Dadi Jaya, Purwodadi District. The farms’ condition in this area was relatively good because dairy cow cooperatives that have bought and collected milk deposited by the community are in existence. Samples from Mojokerto Regency were obtained from independent dairy farms, and the conditions were traditional with minimal maintenance and hygiene management.

Apart from the Malang Regency, the second largest milk producer was Lumajang Regency. The milk samples here were obtained from smallholder farmers in Karangbendo Village, Tekung District, and Kabuaran, Kunir District. These farmers were members of the *Sejahtera* livestock farmer group. The farm condition has implemented good housing management, feed, milking, and health. The farmers produce milk to sell to the central cooperative in Senduro. Another milk sampling location was the Banyuwangi Regency, which was located in the easternmost part of the East Java Province. In this study, samples of dairy cows were taken from Licin and Kalibaru districts (i.e., in the Kaligondo and Ijen Makmur Dairy Cattle Groups). Furthermore, milk samples from Jember Regency were collected from the Rembangan District, the center for dairy cows in Jember and Bondowoso.

### Incidence of SCM and milk sampling

This study collected and analyzed 592 (all quarter) samples from 146 lactating dairy FH cows in 62 farms for SCM prevalence. Milk samples were taken during milking in the morning or evening. In selected areas, all samples were collected from lactating cows aged 3-7 years with parities 1-5 from farms with small (cow’s population, approximately 4-7 heads) to large (cow’s population, >20 heads) commercial farms.

For each regency, the farms with the largest cattle population through stratified random sampling were chosen according to Windria *et al*. [24]. The sampling method is done by milking the cattle (the first and second spurts of milk were discarded), and the third spurts were obtained using a 15-mL conical tube and given a collection code and date. The California mastitis test (CMT) test was performed to detect SCM by taking as much as 1 mL of milk and adding 1 mL of CMT reagent, then placing them into the paddle and homogenizing. The paddle was moved horizontally for 10–20 s, and the reaction results were read on the basis of the mixture change, which is characterized by the shape of the viscous mass with the reaction rate negative, trace, CMT 1 (+), CMT 2 (++), and CMT 3 (+++) [25]. The negative result shows that the mixture did not indicate any viscosity. CMT 1, CMT 2, and CMT 3 were characterized by a slightly thickened mass, thickened mass, and mass formation that resembles gelatin and was challenging to move, respectively. At the cow level, a positive rate was defined by at least a quarter having a positive CMT reaction.

### Survey on management practices

Interviews were conducted with each farmer, and observations of the housing and livestock areas were conducted to validate the interview data. Interviews were performed to identify practical management, cattle attributes (e.g., breed parity, lactation stage, udder cleanliness, mastitis history, housing cleanliness, teat wiping after milking, teat drying after milking, pre-dipping with warm water, and post-dipping with iodine), and farm location conducted by each farmer and then practical management associations with risk factors for SCM were analyzed. The management practices in question include farming experience, ownership of various cows, feed type, feeding management, housing material, reproduction management, hygiene and sanitation, and treatment and prevention against disease. The milking in each area was done twice a day, that is, in the morning (Jember at 01:00 AM; Sidoarjo, Mojokerto, Probolinggo, and Pasuruan at 03:00-04:00 AM; and Malang and Banyuwangi at 04:00-05:00 AM) and evening (Jember at 01:00 PM and others at 03:00-04:00 PM). Jember, Banyuwangi, and some of the farms in Malang use machines in the milking process, and other areas were hand milking. Although the farmers cleaned the cows and cages every day before milking, the hygienic conditions of the house on the observed farms are very varied (clean, moderate, and dirty). On the basis of an observational study of the cows, all cows have normal teat and udder. The procedure of teat wiping with a warm towel or water to stimulate milk let-down and post-dipping with iodine after milking was not performed by all farmers in East Java. For cow nutritional needs, mostly these farmers fed cows twice a day with forage and 1- to 2-kg concentrate (using tofu waste).

### Statistical analysis

The data were qualitatively and quantitatively analyzed. All assembled data were input in Microsoft Excel. The results of the identification of SCM incidents in farmers were processed descriptively and presented in a diagrammatic form. The interview data were tabulated using the IBM SPSS version 26.0 (IBM SPSS Statistics, Chicago, USA), and analyzed using multinomial regression to define the risk factors for the incidence of SCM in East Java (p<0.05 was taken as statistically significant).

## Results

### Farm characteristics and management practices

Management practice from small-scale dairy farming with a subsistence farming system traditionally conducted in East Java. The farmers raise the FH and FH cross-types of dairy cattle and house their cattle in groups. The ownership of cows varies from 3 to 25 heads (average, 8 heads). The number of lactating cows is approximately 1-12 animals (average, 5 heads per farmer). The feeding method are intensive by providing forage and concentrate feed to the cows. In the housing area, all farmers use a rubber base as a floor mat because it is easy to clean and makes the floor not greasy, making it safe for the cows. The feeding practice is the cut-and-carry system, and the grasses are mainly elephant grass (*Pennisetum purpureum*), odot (*P. purpureum* cv. Mott), and field grass. Moreover, no farmer has fodder land. Sometimes, they gave legumes as feed but not every day. Seasonal agriculture waste is also provided to the cattle (e.g., corn leaf and waste; paddy straw). Most farmers provide various concentrates with various amounts and types, usually tofu waste and rice bran with feeding frequency of 2-4 times a day (average, 3 times), with a feeding interval of 12 h. Dairy farmers in East Java prefer to use artificial insemination for their reproduction programs using semen provided by the government.

**Figure-1:**
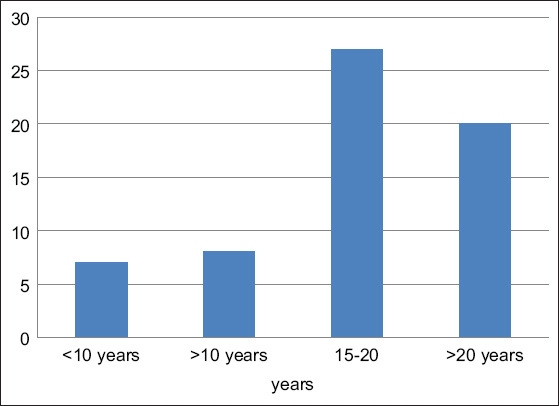
Duration of farming experience on dairy cattle industry.

The cow’s cleanliness in each farm varies (i.e., clean, moderate, and dirty), with cleaning and manure removal done every day before milking (morning and evening). Almost all farmers milk twice a day, particularly at 03:00-06:00 AM and 03:00-04:00 PM. Of the farmers, 96.36% (53/55) and 3.64% (2/55) conducted hand milking and used machines, respectively. Before milking, farmers maintain hygiene and sanitation by washing their hands and cleaning the cows. Moreover, they also wipe the teat or dip the teat in warm water to stimulate milk let-down. The farmers then apply post-dipping using iodine solution after the milking process. Meanwhile, milk production in various East Java farmers widely differs (range, 10-20 L/head/day). The milk prices per liter start from IDR 5250-10,000 (average, IDR 6277). The farmer’s experiences of farming cows are various, and most farmers had 27 years of dairy cattle experience. The distribution of farmer experience time is provided in Figure-1. The farming experience was >20 years, but awareness of good farming practices is still low.

**Figure-2:**
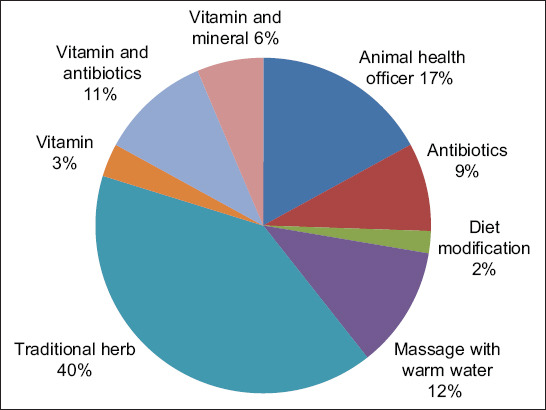
Percentage of subclinical mastitis treatment and prevention perform by farmers.

Mastitis treatment for every farmer is different. The current study found that as many as 25% of farmers use traditional medicine to prevent and treat mastitis. This traditional medicine uses spices, salts, and herbs. Treatment by inviting an animal health officer (16%) and using antibiotics (8%) was conducted when the cows experienced a drastic decrease in milk production or showed clinical mastitis symptoms. The precautionary measure is done by providing vitamin and mineral supplements. The treatment data performed by farmers are presented in Figure-2.

### Prevalence of SCM in East Java

**Figure-3:**
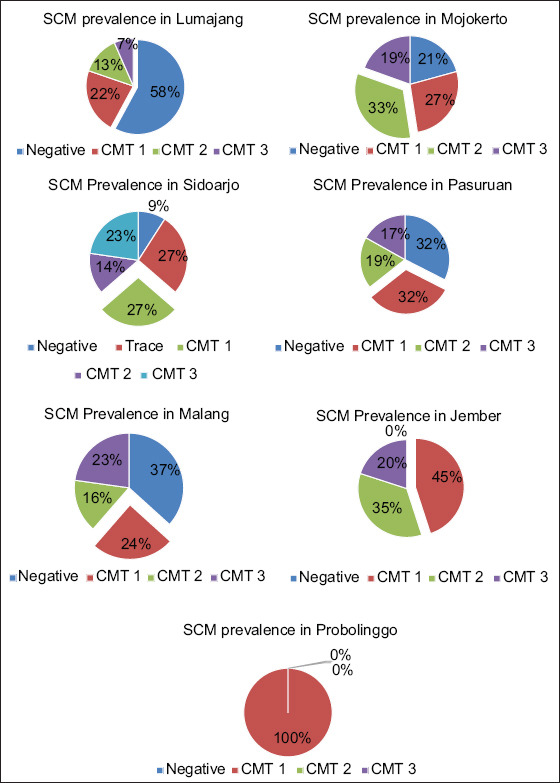
Subclinical mastitis prevalence in East Java.

Research on the prevalence of SCM in East Java shows that the incidence of SCM in East Java province is relatively high with a percentage of 68.18% at the cow level and 66.72% at the quarter level with details of the results of the CMT analysis is presented in Table-1 and Figure-3. Each observed district showed a different prevalence rate ranging from 42% to 100%. Probolinggo district shows the highest prevalence (100% positive SCM), while the lowest is found in Lumajang district (42%). The analysis of the prevalence of SCM for each district is presented in Figure-3. The prevalence in Sidoarjo and Mojokerto is relatively high at approximately 88% and 78%, respectively (Table-1).

**Table-1:** The prevalence of subclinical mastitis in East Java at the quarter level.

CMT	Number	Prevalence (%)
Negative	197	33.28
Total CMT positive	395	66.72
CMT 1	167	28.21
CMT 1	125	21.11
CMT 2	103	17.40
Total	592	100

CMT=California mastitis test

### Relationship between the prevalence of SCM incidence and risk factors in dairy farming in East Java

The CMT investigation results are used as the basis for risk factor analysis to investigate SCM’s association and practical management that farmers performed (Table-2). On the basis of the current study, the incidence of SCM significantly occurred in cows in the early lactation (1-2 months; odds ratio [OR]: 5.671) and mid-lactation (3-6 months; OR: 9.425) than in cows in the late lactation stage (>7 months). Teat wiping after milking with a towel also had a significant association (OR: 42.197). Furthermore, cage cleanliness is better and significantly protects dairy cattle compared with dirty and moderately dirty housing given the impact of the incidence of SCM diseases. Another factor that shows the significance of the risk factor for mastitis incidence is the location (regencies) where the dairy cows are raised. The udder cleanliness, mastitis history, teat drying after milking, pre-dipping and post-dipping showed no significant association with SCM occurrence.

**Table-2:** Risk factor of subclinical mastitis occurrence in East Java Province, Indonesia.

Variabel	Number	Prevalence (%)	p=value	Exp(B)/OR
Lactation stage				
1-2 months	19	3.21	0.000	5.671
3-6 months	248	41.89	0.000	9.425
>7 months	128	21.62	Ref.	
Udder cleanliness				
Bad	64	10.81	0.839	1.073
Good	331	55.91	Ref	
History of Mastitis				
Yes	284	47.97	0.880	0.963
No	111	18.75	Ref	
Teat wiping after milking				
Yes	378	63.85	0.02	42.197
No	17	2.87	Ref	
Teat drying after milking				
Yes	226	38.18	0.854	0.912
No	169	28.55	Ref	
Pre dipping with warm water				
No	253	42.74	0.345	0.667
Yes	142	23.99	Ref	
Post dipping with iodine				
No	150	25.34	0.294	1.442
Yes	245	41.39	Ref.	
Housing cleanliness				
Dirty	12	2.03	0.000	0.120
Moderate	129	21.79	0.009	0.527
Good	254	42.91	Ref	
Location (Regencies)				
Sidoarjo	28	70.09	0.007	0.076
Mojokerto	65	16.45	0.036	0.165
Jember	20	5.06	0.784	1.210
Probolinggo	7	1.77	0.138	3.449
Lumajang	32	8.10	0.544	1.638
Malang	100	25.32	0.784	1.210
Pasuruan	108	27.32	0.609	0.681
Banyuwangi	34	8.61	Ref	

## Discussion

The incidence of SCM in East Java is relatively high, with 68.18% and 66.72% at the cow and quarter levels, respectively. These results indicate a higher prevalence than previous SCM reports on FH cows in Indonesia and Kenya as well as goats in Ethiopia [17,20,26]. In contrast, Hiitiö *et al*. [9] reported that the prevalence of SCM on Holstein, Ayrshire, Finn cattle, Jerseys, and their crossbreeds in Finland decreased from 22.3% to 19.0% from 1991 to 2010. This study’s results were lower than the prevalence of SCM in Nigeria (85.3%) [27], Uganda (86.2%) [28], and Vietnam (88.6%) [29]. The variance in prevalence that occurs among farms may be due to the numerous factors consisting of intrinsic (age, parity, breed, lactation stage, and cows health) and extrinsic (farm management practice, production type, hygiene and sanitation, floor type, and milking method) factors [13]. The disease complexity includes infectious agents that attack the udder and intramammary of cows [26]. Bari and Rahman [30] reported that cases of mastitis on FH and Sahiwal breed can be prevented by conducting routine screening, hygiene management, and disease control improvement. The prevalence of SCM is indeed very high compared with that of clinical mastitis, which only reaches 0.6-18.2% [31]. Adjustment in the dairy industry will also alter the structure and development in the next few years, including dairy farmers’ productivity and economic factors [32].

### Risk factors for SCM in lactating dairy cattle in East Java

The result of this study demonstrates the significance of the SCM prevalence and management practices, including lactation stage, teat wiping after milking, housing cleanliness, and location (Table-2). Significant differences were noted in the impact of the appearance of mastitis diseases. Other management practices that are also significant for SCM are lactation stage, breed, calf suckling, and parity with strong correlation [19]. Decreased milk production can occur in cows identified with SCM in the 1^st^ month of lactation and will continue through the entire lactation period. SCM in the early months of lactation can also increase the risk of death in livestock [33]. Moreover, early lactation (OR=5.671) and mid-lactation (OR=9.425) were found to have an association with SCM. This result is in line with that of Yilma and Atsedemariam [34], who reported that cattle with a lactation stage of< 5 months were almost twice more likely to have SCM than a lactation stage of >8 months (OR: 1.987), and parity 6 showed a risk of 5.9 times more than parities 1-3 (OR: 5.847). The cleaning teat wipe using a towel after milking also has a significant impact on SCM susceptibility (OR:42.197).

However, mastitis history, pre-dipping with warm water, teat drying after milking, and post-dipping with iodine did not significantly affect SCM in dairy cows in East Java. In contrast, a previous study showed that cows with an SCM history and reproductive disorders are more likely significantly associated with SCM prevalence [35,36]. This study showed that analyzing the breed as a factor for all of the farmers raises FH, and this breed has been reported to have a higher OR (OR=5.1) than other breeds [19,35]. The floor material of the cage was also reported to significantly affect the risk of SCM, especially the floor with spilled feed showed a prevalence of 64.2% [34].

The presence of a lesion on the teat also shows a higher SCM score (75.3%) [34]. Sayeed *et al*. [35] reported that the cow’s body condition score (BCS) is significantly associated with the prevalence of SCM, where a greater BCS will produce more milk and is more susceptible to SCM. The process of milking using the hand is known to show a negative association with SCM [37].

The farm locations showed significant associations with SCM. This study found that Sidoarjo and Mojokerto districts were significantly associated with an OR of 0.076 and 0.165, respectively. This occurrence is possible because of the variation in daily temperature where the city with a warmer temperature is more likely to be at risk of having a higher prevalence of SCM. This result is supported by Vitali *et al*. [38], who mentioned that heat load conditions increased the risk of clinical mastitis and have been associated with environmental pathogens. Despite unhygienic milking, the lack of farming management to control mastitis could be a factor in the spread of mastitis-causing bacteria. Thus, paying attention to the procedure of the milking process, particularly from healthy cows and finally from cows indicating SCM, is essential [19]. Moreover, efforts are needed to prevent and reduce mastitis prevalence. A good recording of livestock health, especially regular screening for SCM and udder health, and maintaining personal and equipment hygiene and cleanliness of pens and cows are necessary [39-41]. The designed strategy to reduce SCM prevalence can be made by presenting educational programs and facilities on subclinical and clinical mastitis detection and control methods.

## Conclusion

The prevalence of mastitis in East Java is high (68.18% and 66.72% at the cattle and quarter levels, respectively). The risk factors for mastitis were significantly influenced by the lactation stage, teat wiping after milking, housing cleanliness, and farm locations. This study’s results indicate that the practical management performed by dairy farmers needs to be improved especially on the cleanliness of cages and cows to reduce the prevalence of SCM to increase dairy cow productivity and also prevent SCM incidents in East Java, Indonesia.

## Authors’ Contributions

HK and DCW: Formulated the presented idea, originated the study, developed the theory, supervised the data collection, and wrote the manuscript. HBS and RY: Carried the data collection, field observation, CMT analysis, and co-wrote the manuscript. HK conducted the statistical analysis. All authors discussed the results and contributed to the final manuscript.
